# Marine and giant viruses as indicators of a marine microbial community in a riverine system

**DOI:** 10.1002/mbo3.392

**Published:** 2016-08-09

**Authors:** Lisa M. Dann, Stephanie Rosales, Jody McKerral, James S. Paterson, Renee J. Smith, Thomas C. Jeffries, Rod L. Oliver, James G. Mitchell

**Affiliations:** ^1^ School of Biological Sciences at Flinders UniversityAdelaideSouth AustraliaAustralia; ^2^ Department of MicrobiologyOregon State UniversityCorvallisOregonUSA; ^3^ School of Computer ScienceEngineering and MathematicsFlinders UniversityAdelaideAustralia; ^4^ Hawkesbury Institute for the EnvironmentWestern Sydney UniversityPenrithNew South WalesAustralia; ^5^ Land and Water Research Division at the Commonwealth Scientific and Industrial Research Organisation (CSIRO)AdelaideSouth AustraliaAustralia

**Keywords:** giant viruses, marine‐freshwater transitions, metagenomics, riverine systems, viral ecology, viruses

## Abstract

Viral communities are important for ecosystem function as they are involved in critical biogeochemical cycles and controlling host abundance. This study investigates riverine viral communities around a small rural town that influences local water inputs. Myoviridae, Siphoviridae, Phycodnaviridae, Mimiviridae, Herpesviridae, and Podoviridae were the most abundant families. Viral species upstream and downstream of the town were similar, with *Synechoccocus phage*,* salinus*,* Prochlorococcus phage*,* Mimivirus A,* and *Human herpes 6A virus* most abundant, contributing to 4.9–38.2% of average abundance within the metagenomic profiles, with *Synechococcus* and *Prochlorococcus* present in metagenomes as the expected hosts for the phage. Overall, the majority of abundant viral species were or were most similar to those of marine origin. At over 60 km to the river mouth, the presence of marine communities provides some support for the Baas‐Becking hypothesis “*everything is everywhere*, but, *the environment selects*.” We conclude marine microbial species may occur more frequently in freshwater systems than previously assumed, and hence may play important roles in some freshwater ecosystems within tens to a hundred kilometers from the sea.

## 1 Introduction

Aquatic viral communities are important for ecosystem function as viral‐mediated lysis of hosts accentuates nutrient and dissolved organic matter release, modifies key processes such as primary production, facilitates genetic exchange between microorganisms and controls bacterial community composition and abundance (Fuhrman, 1999; Newton, Jones, Eiler, McMahon, & Bertilsson, 2011; Suttle, 2005; Wilhelm & Matteson, 2008; Wommack & Colwell, 2000). Previously, transitions of species between marine and freshwater environments was considered rare, with studies showing genetic distinctness of viruses between freshwater and other aquatic environments, indicating specificity in freshwater viromes at the community scale (Roux et al. 2012). Transitions between marine and freshwater environments are considered rare due to a lack of close relatedness between macro‐ and micro‐organisms in marine and freshwater environments (Logares et al., 2009). However, there is evidence for viral propagation in different biomes. For instance, Sano, Carlson, Wegley, and Rohwer (2004) showed phage communities from lake water could replicate when incubated with marine microbes, suggesting either broad host ranges allow phage to move between biomes or their microbial hosts are able to move and grow within different biomes. In addition, Wilhelm et al. (2006) found that marine *Synechococcus* cyanophage were abundant in a Laurentian great lake and suggested these marine phage may be important to freshwater systems. Wilhelm et al. (2006) suggested two mechanisms for the transport of the marine phage species, either via boat ballast water, or through the presence of a natural host.

Alternatively, the occurrence of marine viruses in freshwater systems could relate to the historical conditions of the environment, with the microbial community serving as a biomarker of past conditions. The Murray Basin and its rivers formed during the Cainozoic era, after the separation of Antarctica and the southern border of Australia (Kingham, 1998). During the Oligocene to mid‐Miocene, a substantial marine transgression occurred which led to the seas flooding the eastern coast of South Australia (Brown, Campbell, & Crook, 1968; Kingham, 1998). Following this, several marine transgressions and regressions occurred during the upper Miocene to Pliocene (Brown et al., 1968; Kingham, 1998). From these historical events, fossil records of marine organisms exist within the sediments and limestone underlying the Murray River in this region, and large concentrations of salt remain within the sediments and soil (Kingham, 1998; Zhisheng, Bowler, Opdyke, Macumber, & Firman, 1986). These regions contain a large number of active and inactive fossil salt‐lake systems (Bowler & Magee, 1978; Zhisheng et al., 1986). The high concentration of salt underlying the Murray River has led to salinity problems due to discharge of saline ground waters (Brown & Stephenson, 1991). These highly saline groundwaters may host species typically observed in saline environments and therefore introduce these microbes into the freshwater river system.

Viruses are highly dispersed but local environmental conditions will enrich specific viral species via selective pressures (Angly et al., 2006). Viral species that are adapted to managing a range of selective pressures will be able to persist throughout the system. Persistence of bacterial genotypes was identified previously within the same river system and was suggestive of generalists that can adapt to system fluctuations (Dann, Smith, Jeffries, et al., 2016b). To our knowledge, no such study looking at the temperate viral communities in a fluvial system exists. Therefore, this study works to bridge this gap by investigating the whole viral community and the potential shifts in this community profile in the Murray River upstream and downstream of the small rural town of Murray Bridge in South Australia. The aims of this study are to describe the viral community within a fluvial system and assess its functional characteristics, determine whether the viral community is stable over a river reach of several kilometers despite changing conditions, and lastly, determine what viral types were introduced by a township and whether this greatly influenced the viral community. We hypothesize a higher abundance of human‐ and/or animal‐associated viruses downstream due to the potential anthropogenic impact of water runoff. To test this hypothesis, viral metagenomics was performed to identify the diversity of freshwater viral communities upstream and downstream.

## 2 Experimental Procedures

### 2.1 Sample sites

Samples were collected 3.3 km upstream from Murray Bridge at Mobilong (−35.099, 139.289), and 3.3 km downstream from Murray Bridge at Long Island reserve, South Australia (−35.131, 139.299) on February 11th, 2014. Murray Bridge is located approximately 60 km upstream from the river mouth. Triplicate samples were collected at each site for silica, nitrate, nitrite, ammonium, phosphate, and iron concentration measurements. Nutrient concentrations were measured using a LF 2400 photometer and Aquaspex water testing products. To test whether nutrient concentrations were significantly different between sample sites, a two‐tailed student *t*‐test with assumed unequal variance was performed. The downstream site had an island separating the river, whereas the upstream site was an open water site with minimal vegetation. According to monitoring station data, the water level was 0.55 m AHD, water temperature was 24.1°C, electrical conductivity was 305 μs/cm and water flow rate was 0.09 m/s at the time of sampling (DEWNR, 2014a,b). The sampling sites were characterized by slow water flow rates and hence were highly turbulent (Dann et al. 2016a).

### 2.2 Virus‐like particle (VLP) and prokaryote enumeration

Six triplicate samples were collected mid‐water column and from the river bank at each sampling site for VLP and prokaryote enumeration via flow cytometry. Samples were collected according to Dann et al. (2014). Briefly, 1 ml sample aliquots were placed into 2 ml cryovials containing 20 μl of glutaraldehyde (0.5% final concentration) and stored at 4°C for 15 min in the dark before being snap frozen in liquid nitrogen and stored at −80°C (Brussaard, 2004; Dann et al., 2014). Flow cytometry was performed within 3 weeks to avoid sample deterioration (Brussaard, 2004).

This study adopts the same flow cytometry protocol as previous studies (Dann et al., 2014; Dann, Paterson, Newton, Oliverand, & Mitchell, 2016a; Dann, Smith, Jeffries, et al., 2016b; Roudnew et al., 2012; Seymour, Seuront, Doubell, & Mitchell, 2008; Smith, Paterson, Sibley, Hutson, & Mitchell, 2015). Briefly, thawed samples were diluted 1:100 with Tris‐EDTA buffer (pH 7.4, 0.2 μm filtered, 10 mmol L^−1^ Tris, 1 mmol L^−1^ EDTA) and stained with SYBR Green I (1:20,000 final dilution; Molecular Probes). Samples were incubated at 80°C for 10 min in the dark to optimize VLP counts (Brussaard, 2004; Brussaard, Marie, & Bratbak, 2000; Dann et al., 2014; Dann, Peterson, et al., 2016a; Dann, Smith, et al. 2016b; Marie, Brussaard, Thyrhaug, Bratbak, & Vauolt, 1999; Marie, Partensky, Jacquet, & Vaulot, 1997; Schapira et al., 2009; Seymour, Seuront, & Mitchell, 2007). Fluorescent beads of 1 μm diameter (final concentration of approximately 10^5^ beads ml^−1^, Molecular Probes) were added to each sample and served as an internal concentration and size standard. Measured flow cytometry parameters were normalized to fluorescence and bead concentration (Brussaard, 2004; Brussaard et al., 2000; Dann et al., 2014; Gasol & Del Giorgio, 2000; Marie et al., 1997, 1999; Schapira et al., 2009).

A FACSCanto II flow cytometer (BD) equipped with red (633 nm, 17 mW), blue (488 nm, 20 mW, air‐cooled), and violet (405 nm, 30 mW) lasers and utilizing a phosphate‐buffered saline (PBS) sheath fluid was employed. Green fluorescence (SYBR I), forward‐angle light scatter (FSC), and right‐angle side scattered light (SSC) were acquired for each sample. Samples were run for 2 min on a low flow rate setting to record <1000 events per second. Triplicate negative control samples containing Tris‐EDTA buffer (0.2 μl filtered) stained with SYBR Green I were analyzed during each session to eliminate potential background noise from flow cytometer artifacts or sample preparation (Dann et al., 2014). Resulting cytograms and histograms were exported as FSC 3.0 files and used for VLP subpopulation enumeration via FlowJo (Tree Star, Inc.) (Smith et al., 2015). VLP populations were discriminated by side‐scatter and SYBR Green fluorescence (Brussaard, 2004; Dann et al., 2014; Dann, Peterson, et al., 2016a; Dann, Smith, et al. 2016b; Marie et al., 1997, 1999).

### 2.3 DNA concentration, purification, and sequencing

For metagenomic analysis, a total of 30 L of water was collected mid‐water column from the river bank at each sampling site using sterile carboys. While sampling, care was taken not to disrupt the river bed as water column samples were desired. Samples were processed immediately, firstly by prefiltering through 5 μm filters (Whatman) to remove large suspended particulate matter using a series of vacuum pumps and Nalgene filtering units. FeCl_3_ precipitation was employed for viral concentration following the methods by John et al. (2011), Hurwitz, Deng, Poulos, and Sullivan (2013a), and Hurwitz, Hallam, and Sullivan (2013b) with adjustments to freshwater systems. Namely, the concentration of FeCl_3_ added to the filtrate was amended to 10 mg of Fe per liter of river water, as used in previous freshwater systems (Chang, Stevenson, Bryant, Woodward, & Kabler, 1958; Zhu, Clifford, & Chellam, 2005), to enable proper precipitation of freshwater viruses. Also, additional filtering steps were employed due to the presence of fine suspended particles. Briefly, 30 L pre‐filtered samples were filtered through a series of filters, Whatman GF/A glass microfiber filters (1.6 μm retention; 150 mm diameter), Whatman GF/F glass microfiber filters (0.7 μm retention; 150 mm diameter), and Millipore express filters (0.22 μm retention; 142 mm diameter). FeCl_3_ was added to the viral filtrate at a concentration of 10 mg of Fe per liter of river water. Viral particles were left to precipitate at room temperature for 1 hr with constant mixing. The viral precipitate solution was filtered through polycarbonate membrane filters (1.0 μm retention; 142 mm diameter) on top of Pall Supor filters (0.8 μm retention; 142 mm diameter) to collect the viral precipitate. All viral filtering steps were carried out using 142 mm acrylic in‐line filtering towers (ENVCO). Viral concentrations were determined via flow cytometry at each step of the filtering process to ensure sufficient concentration. A negative control of sterile autoclaved MilliQ water was run through the filtering process and analyzed via flow cytometry to ensure method sterility. The filters containing the viral precipitate were then treated with magnesium‐EDTA‐ascorbate buffer (0.1 mol L^−1^ Mg_2_EDTA, 0.2 mol L^−1^ ascorbic acid, pH 6.0) to resuspend the viral particles and purified using DNase I (100 U ml^−1^) in reaction buffer (10 m mol L^−1^ Tris‐HCl pH 7.6, 2.5 m mol L^−1^ MgCl_2_, 0.5 m mol L^−1^ CaCl_2_) and left on a tube rotator for 2 hr at room temperature until extraction. Extraction was carried out using a QIAamp MinElute virus spin kit (Qiagen Pty. Ltd.) as per the manufacturer's instructions (Fig. S7).

To acquire adequate genomic material for sequencing GenomiPhi was used for DNA amplification (GE Healthcare) which created nonspecific amplification via polymerase phi29 (Robin et al., 2012). As we were interested in the differences between sampling sites rather than absolute taxonomic abundances, the potential biases introduced from this method were not considered problematic as they would be present in all the samples. Genomic DNA was sequenced using Illumina MiSeq 2 x 250 bp sequencing (Molecular Research). Briefly, genomic DNA was isolated, purified, fragmented, and ligated to sequencing adapters. Once amplification and denaturation was performed, libraries were prepared via Nextera DNA Sample Preparation Kit (Illumina) to produce individual barcode indices. Each library was prepared using 50 ng of DNA per sample. The libraries were then pooled and sequenced and an Experion Automated Electrophoresis Station (Bio‐Rad) was then used to determine the insert sizes for each library. Insert sizes ranged from 300 to 850 bp, with an average size of 500 bp. Pooled 12 pM libraries were then loaded to a 500 Cycles v3 Reagent cartridge (Illumina) and sequenced using Illumina MiSeq.

### 2.4 Taxonomic analyses

Sequenced viral DNA in FASTQ format were quality filtered, trimmed and adapters, unknown terminal bases, poly‐A tails, and low‐quality 3′ read regions were removed via FqTrim (Pertea, 2015). Paired‐ends were joined and RiboPicker was used to remove 16s, 18s, 28s, and 5.8s ribosomal RNA to increase the quality of sequences and relevance of the results (Schmieder, Lim, & Edwards, 2012). Bowtie 2 was used to remove human sequences using the *H. sapiens* UCSC hg18 Bowtie 2 index (Deng et al., 2015; Langmead & Salzberg, 2012). Sequences were dereplicated for 100% sequence similarity using USEARCH (Edgar, 2010). Dereplicated sequences were assembled using Velvet, with a k‐mer length of 49 (Zerbino & Birney 2008). These contigs were then analyzed via tBLASTx with the NCBI viral RefSeq database using an e‐value of 10^−7^ (Deng et al., 2015). Viral taxonomic representation was determined via Galaxy (Blankenberg et al., 2010; Giardine et al., 2005; Goecks, Nekrutenko, & Taylor, 2010). Velvet was employed for assembling due to its success with previous Illumina viral metagenomics datasets showing highly reliable contig construction using short read sequencing, as well as its compatibility with the file formats obtained from postprocessing (Vázquez‐Castellanos, García‐López, Pérez‐Brocal, Pignatelli, & Moya, 2014). VelvetOptimiser was employed to determine optimum assembly parameters. Hash values from 20 to 399 were explored with a k‐mer length of 49 chosen to allow a balance between specificity and sensitivity. The short paired fasta file option was used with a minimum contig length of 100 and a coverage cut‐off value of 15×. Velvet was selected over MetaVelvet as no significant difference was found between them (Vázquez‐Castellanos et al., 2014).

For bacterial sequence annotation, each metagenome was uploaded to the MetaGenome Rapid Annotation with Subsystem Technology (MG‐RAST) online server v3.6 (http://metagenomics.nmpdr.org/) and compared to the SEED nonredundant database via BLASTX with a minimum alignment length cutoff of 50 bp and minimum sequence identity cutoff of 65% (Meyer et al., 2008). Only sequence hits with an E‐value of <10^−5^ were considered to be significant for further analyses. The number of sequences classified were normalized to the metagenome sequence size to allow comparison between the upstream and downstream metagenome. For functional annotation, MG‐RAST was used to categorize gene sequences into general metabolic systems and SEED subsystems using the aforementioned parameters. These sequence data have been submitted to the GenBank database under accession number SAMN04631800.

### 2.5 Data analysis

Taxonomic and metabolic profiles were exported to the Statistical Analysis of Metagenomic Profiles (STAMP) software package v2.1.3 to compare differences in taxonomy and metabolism between the upstream and downstream metagenomes (Parks & Beiko, 2010). Statistically significant differences were calculated using Fisher's exact test with Benjamini–Hochberg false‐discovery‐rate (FDR) multiple test correction. The FDR method was used to calculate false positive percentage, reported in extended error bar plots as *q* values. The *q* value threshold was >.05 and the *p*‐value threshold was.05. The 95% confidence intervals were calculated using the Newcombe–Wilson method.

Rank abundance graphs were constructed of the lowest classification sequence reads to assess whether viral diversity displays power law behavior (Edwards & Rohwer, 2005). For rigor, maximum likelihood estimation was used to fit a power law and test significance (Clauset, Shalizi, & Newman, 2009). Specifically, an optimal power law (*y* = *Ax* ^−α^) was found for *n* species, counting from the most to least abundant, for each site (Clauset et al., 2009). The single most abundant genus was removed as an outlier, and the model fitted to genera of equal or higher abundance than xmin. The resulting model was tested for significance via a Kolmogorov–Smirnov (KS) goodness of fit test. If the data and model fit the same distribution, the KS test statistic *D*_*n*_ is less than the KS critical value (95% confidence).

## 3 Results

### 3.1 VLP and prokaryote abundance

Flow cytometric analysis revealed two VLP subpopulations, VLP 1 and VLP 2, and two prokaryotic subpopulations, low DNA (LDNA) and high DNA (HDNA), via monoparametric histograms of SYBR Green I fluorescence and biparametric cytograms of side‐scatter (SSC) and SYBR Green I fluorescence (Fig. S1). Table 1 shows the average abundance of VLP and prokaryote subpopulations. Two‐sample *t*‐testing showed no significant difference between the upstream and downstream mean prokaryotic or VLP abundances (*p* ≥ .13).

**Table 1:** Average abundance of VLP and prokaryotic subpopulations as determined via flow cytometric enumeration

Subpopulation	Average abundance particles/cells ml^−1^ (95% CI, *n*)	
	Upstream	Downstream
VLP 1	1.5 x 10^6^ (1.7 x 10^6^, 3)	4.2 x 10^5^ (2 x 10^5^, 3)
VLP 2	7.2 x 10^5^ (6.3 x 10^5^, 3)	4.9 x 10^4^ (6.6 x 10^4^, 3)
Total VLP	2.2 x 10^6^ (2.3 x 10^6^,6)	4.6 x 10^5^ (2.4 x 10^5^, 6)
LDNA	3.3 x 10^7^ (1.2 x 10^7^, 3)	2.3 x 10^7^ (5.8 x 10^6^, 3)
HDNA	1.6 x 10^7^ (6.2 x 10^5^, 3)	1.1 x 10^7^ (4.7 x 10^6^, 3)
Total prokaryotes	5.0 x 10^7^ (1.2 x 10^7^, 3)	3.4 x 10^7^ (2.9 x 10^7^, 3)

### 3.2 Nutrient concentrations

Table 2 shows the nutrient concentrations upstream and downstream. Nitrate and phosphate concentrations were significantly higher at the upstream site (*p* < .009, *p* < .045) via a two‐tailed Student's *t*‐test with assumed unequal variance.

**Table 2:** Mean nutrient concentrations upstream and downstream

Nutrient	Concentration (mg L^−1^) (95% CI, *n*)	
	Upstream	Downstream
Phosphate	0.83 (0.24, 3)	0.33 (0.24, 3)
Silica	0.97 (0.07, 3)	0.87 (0.26, 3)
Nitrite	0.02 (0.01, 3)	0.01 (0, 3)
Nitrate	0.93 (0.13, 3)	0.37 (0.17, 3)
Iron	0.20 (0.11, 3)	0.27 mgL^−1^ (0.17, 3)
Ammonium(total NH_3_/total NH^4+^)	0.04 (0, 3)	0.07 (0.04, 3)

### 3.3 Taxonomic profiling of riverine viral metagenomes

Raw unjoined FASTQ files contained 2,504,235 and 2,078,210 sequences for the upstream site and 2,626,578 and 2,228,065 sequences for the downstream site. Quality filtering via FqTrim removed 19% and 37% of the upstream sequences and 26% and 32% of the downstream sequences, whereas Ribopicker removed 942 sequences from the upstream sample and 285 sequences for the downstream sample. Bowtie 2 revealed 0.02% human contamination in both sample site metagenomes, with 174 sequences removed for the upstream sample and 269 sequences removed for the downstream sample. Dereplication via USEARCH led to the removal of <32 sequences for the upstream and downstream samples.

#### 3.3.1 Upstream

At the upstream site 262 viral species were identified. At the family level, the average abundance of Myoviridae was 30.3%, Siphoviridae was 23.3%, Mimiviridae was 15.1%, Phycodnaviridae was 12.1%, Herpesviridae was 8.8%, Podoviridae was 7.2%, and Microviridae was 1.3%, respectively (Fig. S2).

At the genus level, the average abundance of T4‐like virus was 26.4%, Mimivirus was 19.4%, Roseolovirus was 16.6%, Chlorovirus was 8.0%, Prasinovirus was 6.0%, Coccolithovirus was 5.4%, Prymnesiovirus and Microvirus were 2.9%, and Varicellovirus was 2.3%, respectively (Fig. S3).

**Figure 1:**
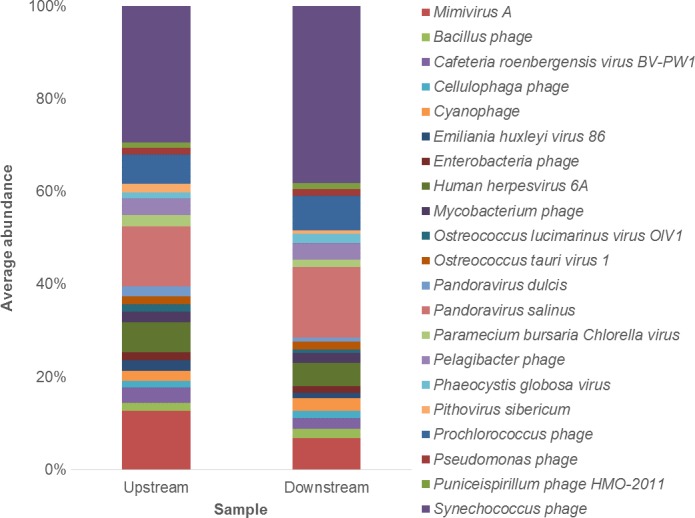
Average abundance of viral species upstream and downstream. For clarity, only genera representing ≥0.5% average abundance are shown. Viral species determined via 49‐kmer contigs blasted against the NCBI nucleotide database using tBLASTx

At the species level, the average abundance of *Synechoccocus phage* was 29.4%, *Pandoravirus salinus* was 12.9%, *Mimivirus* A was 12.7%, *Human herpes 6A virus* and *Prochlorococcus phage* were 6.4%, *Pelagibacter phage* was 3.7%, *Cafeteria roenbergensis virus* was 3.4%, and *Paramecium bursaria chlorella virus* was 2.4%, respectively (Fig. 1).

#### 3.3.2 Downstream

At the downstream site 274 viral species were identified. At the family level, the average abundance of Myoviridae was 33.5%, Siphoviridae was 31.7%, Phycodnaviridae was 10.4%, Mimiviridae was 8.6%, Podoviridae was 7.1%, Herpesviridae was 6.4%, and Coccolithovirus was 3.6% (Fig. S2).

At the genus level, the average abundance of *T4likevirus* was 37.9%, *Roseolovirus* was 16.7%, *Chlorovirus* was 7.6%, *Prasinovirus* was 6.6%, *Prymnesiovirus* was 6.4%, and *Mimivirus* was 5.6%, respectively (Fig. S3).

At the species level, the average abundance of *Synechoccocus phage* was 38.2%, *Pandoravirus salinus* was 15.2%, *Prochlorococcus phage* was 7.5%, *Mimivirus A* was 6.8%, *Human herpes 6A virus* was 4.9%, *Pelagibacter phage* was 3.6%, and *Cyanophage* was 2.7%, respectively (Fig. 1). The most abundant *Synechoccocus phage* strains were *S‐SKS1, S‐PM2. S‐CBS4, S‐SM2,* and *S‐SSM7*. The most abundant *Prochlorococcus phage* type strains were *P‐SSM2, Syn1,* and *P‐SSM7*.

### 3.4 Rank abundance

**Figure 2:**
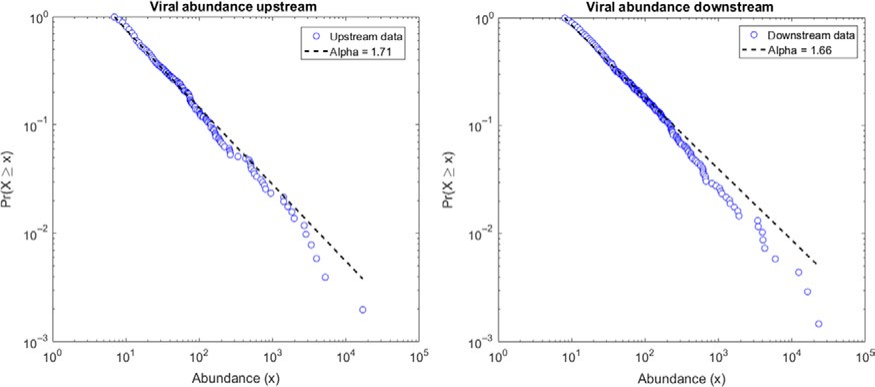
Rank abundance graphs of viral genotypes (A) upstream and (B) downstream. Genotypes determined using 49 k‐mer contigs against the NCBI viral nucleotide database using tBLASTx

Rank abundance graphs of the viral genotypes upstream and downstream revealed a generalized Pareto distribution with no significant difference in function and slope between the sampling sites (Fig. 2). The slope was −1.71 upstream and −1.66 downstream (Fig. 2 and Table S1).

### 3.5 Bacterial metagenomic profile

The best hit classification revealed 363 known hits out of 57,695 sequences upstream, and 357 known hits out of 41,609 sequences downstream. The bacterial profile revealed the most abundant bacterial phyla were Chloroflexi, Proteobacteria, and Actinobacteria, which contributed to 51.8%, 20.1%, and 20.6% average abundance upstream and 51.8%, 23.1%, and 15.4% average abundance downstream (Fig. S4). Bacteroidetes, Firmicutes, and Cyanobacteria were also present, contributing to 3.2%, 1.7%, and 1.2% average abundance upstream and 2.8%, 2.8%, and 2.0% average abundance downstream (Fig. S4).

At the family level, Chloroflexaceae, Herpetosiphonaceae, and Burkholderiaceae were the most abundant, accounting for 47.1%, 23.5%, and 7.6% of the average abundance upstream and 44.4%, 25.2%, and 11.2% of the average abundance downstream (Fig. S5). Streptomycetaceae and Nocardiodaceae were also abundant upstream, contributing to 3.8% and 2.9% average abundance, whereas Propionibacteriaceae and Myxococcaceae were abundant downstream, contributing to 2.5% and 2.2% average abundance (Fig. S5).

At the genus level, *Chloroflexus, Herpetosiphon,* and *Polynucleobacter* were abundant, contributing to 43.3%, 21.6%, and 5.5% average abundance upstream and 43.7%, 24.9%, and 7.3% average abundance downstream (Fig. S6). *Streptomyces* was also abundant upstream, accounting for 3.5% average abundance, whereas *Propionibacterium* was abundant downstream, accounting for 2.5% average abundance (Fig. S6).

**Figure 3:**
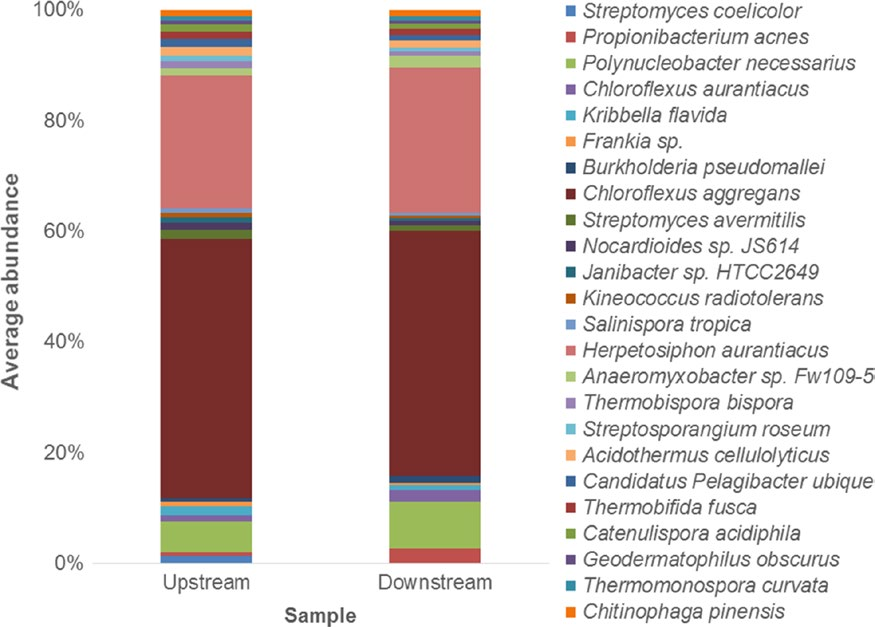
Average abundance of bacterial species upstream and downstream. For clarity, only species representing ≥1.0% total average abundance are shown. Bacterial species determined via the MG‐RAST server using the SEED nonredundant database

At the species level, *Chloroflexus aggregans, Herpetosiphon aurantiacus,* and *Polynucleobacter necessarius* were the most abundant species and accounted for 46.8%, 24.0%, and 5.5% average abundance upstream and 44.3%, 26.4%, and 8.5% average abundance downstream (Fig. 3). *Propionibacterium acnes, Chloroflexus aurantiacus,* and *Anaeromyxobacter* were also abundant downstream, accounting for 2.6%, 2.1%, and 2.0% average abundance, respectively (Fig. 3).

Bacterial species present at lower average abundances upstream were *Acidothermus cellulolyticus* (1.8%)*, Kribbella flavida* (1.6%)*, Streptomyces avermitilis* (1.6%)*, Streptomyces coelicolor* (1.4%)*, Nocardioides sp. JS614* (1.4%)*, Candidatus pelagibacter ubique* (1.4%)*, Thermobifida fusca* (1.3%), and *Thermobispora bispora* (1.2%) (Fig. 3). Other bacterial species present, but at lower average abundances downstream, were *Burkholderia pseudomallei* (1.3%)*, Acidothermus cellulolyticus* (1.3%)*, Candidatus pelagibacter ubique* (1.1%)*, Thermobifida fusca* (1.0%)*, Catenulispora acidiphila* (1.1%), and *Chitinophaga pinensis* (1.2%) (Table S2).

The marine bacterial species present upstream were *Synechococcus CC9605* (0.9%) and *CC9311* (1.3%)*, Marinobacter hydrocarbonoclasticus* (0.4%), *marine Actinobacterium PHSC20C1* (0.2%), *Bacteriovorax marinus* (0.2%), *Idiomarina baltica* (0.1%), and *Idiomarina loihiensis* (0.1%) (Table S2). Marine bacterial species present downstream were *Prochlorococcus marinus* (0.11%), *Synechococcus CC9605* (0.6%) and *CC9311* (1.0%)*, Bacteriovorax marinus* (0.3%), *marine Actinobacterium PHSC20C1* (0.4%) and *Marinobacter hydrocarbonoclasticus* (0.3%), *Idiomarina baltica* (0.1%), and *Idiomarina loihiensis* (0.1%) (Table S2).

### 3.6 Functional analyses

**Figure 4:**
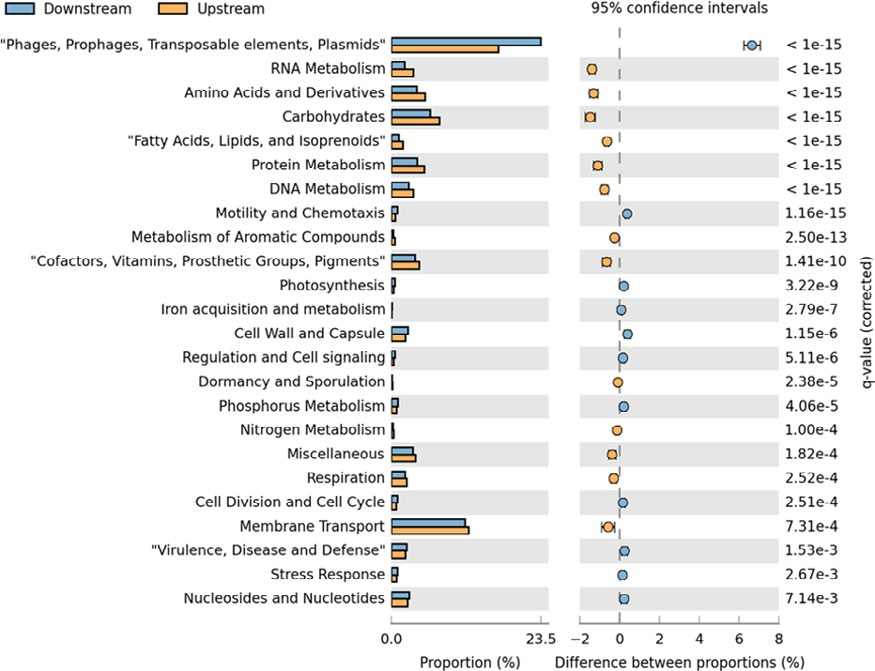
Extended error bar plot comparing the functional classification into SEED subsystems at level 1 for upstream and downstream metagenome sequences. Subsystems were determined via the MG‐RAST server using the SEED nonredundant database

Functional subsystem analysis revealed 2,751 subsystems were characterized upstream, whereas 2,637 subsystems were characterized downstream. The most abundant function at level 1 classification was related to phage, prophage, transposable elements, and plasmids, which accounted for 16.8% of the sequences upstream and 23.5% of the sequences downstream (Fig. 4). Membrane transport, carbohydrates, amino acids, and derivatives and protein metabolism were also abundant, contributing to 12.2%, 7.6%, 5.3%, and 5.2% of the sequences upstream and 11.6%, 6.1%, 4.0%, and 4.1% of the sequences downstream (Fig. 4). There was a significantly higher proportion of sequences attributed to phage, prophage, transposable elements, and plasmids found downstream (Fig. 4).

**Figure 5:**
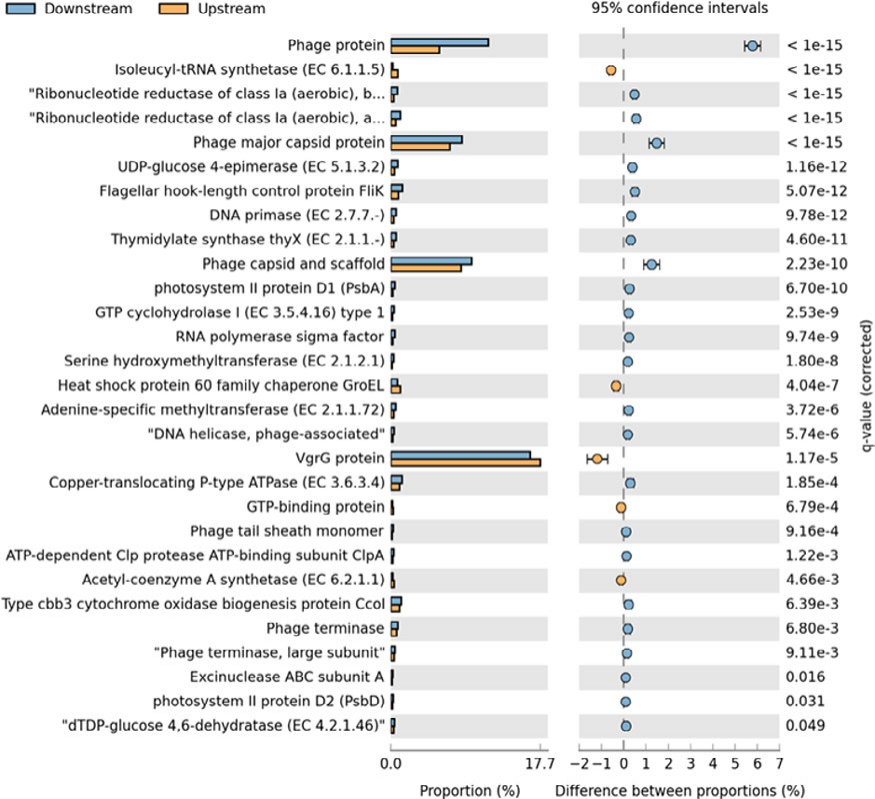
Extended error bar plot comparing the functional classification into SEED subsystems at the functional level for upstream and downstream metagenome sequences. Subsystems were determined via the MG‐RAST server using the SEED nonredundant database. For clarity only functions with ≥80 sequences assigned to them are shown

At the functional level, the most abundant functions were phage‐related, such as VgrG protein, which contributed to 17.7% of the sequences upstream and 16.6% of the sequences downstream (Fig. 5). In addition, phage protein, phage capsid and scaffold and phage major capsid protein contributed to 5.8%, 8.4%, and 7.0% of the sequences upstream and 11.6%, 9.6%, and 8.5% of the sequences downstream (Fig. 5). Flagellar hook‐length control protein FliK, copper translocating P‐type ATPase, type cbb3 cytochrome oxidase biogenesis protein Ccol, and ribonucleotide reductase of class Ia (aerobic) alpha subunit were also abundant, contributing to 0.9%, 1.1%, 1.0%, and 0.6% of the sequences upstream and 1.4%, 1.4%, 1.3%, and 1.1% of the sequences downstream (Fig. 5). Phage protein was significantly higher downstream (Fig. 5).

## 4 Discussion

### 4.1 Viral abundance

Typically, viral abundances are higher than prokaryotic abundance, however, the opposite results were found in this study (Table 1). Viral abundances ranged from 4.9 × 10^4^ to 2.2 × 10^6^ particles ml^−1^, which is low compared to previous reports of viral abundance ranging from 4.1 × 10^7^ to 2.5 × 10^8^ particles ml^−1^ within freshwater systems (Maranger & Bird, 1995). Viral abundances in this study were also low compared to Dann, Peterson, et al. (2016a), where the same river system was sampled and showed viral abundances ranging from 1.7 to 6.7 × 10^7^ particles ml^−1^ (Dann, Peterson, et al. (2016a)). While VLP abundance studies within the Murray River system by Dann, Smith, Tobe, et al. (2016c) found viral abundances ranging from 2.5 × 10^5^ to 2.4 × 10^7^ particles ml^−1^, which were more similar to the abundances observed in this study. Dann, Peterson, et al. (2016a) and Dann, Smith, Tobe, et al. (2016c) sampled during winter, whereas this study sampled during summer, when higher viral abundances are expected (Bettarel, Sime‐Ngando, Amblard, Carrias, & Portelli, 2003; Ma, Sun, Mao, Yu, & Wang, 2013). A potential explanation could be solar radiation levels causing lower viral abundance during summer due to higher viral decay and degradation (Ma et al., 2013).

The similar viral abundance ranges in this study and Dann, Smith, Tobe, et al. (2016c), which sampled during the same year, may be due to the delivery of environmental water during 2013 and 2014, via flows and releases from locations upstream, resulting in higher flows to the South Australian portion of the Murray River sampled here (DEWNR, 2014a,b; Department of Primary Industries (DPI), 2014).This potentially indicates that due to the highly regulated water flows in this river, the similar abundances may be due to annual flow changes throughout this fluvial system impacting VLP population input and removal. Therefore, VLP abundance may be more dependent on annual river flow dynamics rather than typical seasonal variations.

### 4.2 Viral diversity upstream and downstream

Comparisons of the viral species upstream and downstream revealed lower diversity upstream, indicating the potential input of viral species with transport downstream. However, despite this, diversity comparisons showed an overall similarity in species upstream and downstream with the primary difference between sites being species abundance (Fig. 1). For instance, *Human herpes 6A* and *Mimivirus A* were more abundant upstream, whereas *Synechocococcus phage* and *Pandoravirus salinus* were more abundant downstream (Fig. 1).

*Human herpes 6A* infects the majority of the human population and exhibits latency within its host with epidemics of *Human herpes 6* often occurring during summer months, which was when sampling occurred in this study (Arbuckle & Medveczky, 2011; Freitas, Monteiro, & Linhares, 2000). The abundance of *Human herpes 6A* in this river system is potentially due to the dense human population in contact with this location of the Murray River. The higher abundance of *Human herpes 6A* upstream may be due to the presence of houseboats or caravan parks near the upstream site.

*Mimivirus A* was more abundant upstream (Fig. 1), which may be attributed to a higher abundance of *Acanthamoeba* hosts (Moliner, Fournier, & Raoult, 2010). *Synechococcus phage* was more abundant downstream (Fig. 1), and was associated with a higher abundance of *Synechococcus RS9917* downstream (Table S1). *Pandoravirus salinus* was more abundant downstream (Fig. 1). As this giant virus also infects *Acanthamoeba* but is marine in origin (Philippe et al., 2013), its higher abundance downstream may be related to an abundance of *Acanthamoeba* in addition to higher salinity levels.

### 4.3 Bacterial diversity upstream and downstream

Comparisons of the bacterial species upstream and downstream also revealed a similarity in species (Fig. 3). *Chloroflexus aggregans, Herpetosiphon aurantiacus,* and *Polynucleobacter necessarius* were abundant at both sampling sites (Fig. 3). As *C. aggregans* is a thermophilic, phototrophic species capable of gliding motility that is able to form dense cell aggregates rapidly, its persistence in the system may be due to its ability to form biofilms on the river bed, which may have been resupplied from these benthic sources into the planktonic portion (Hanada, Shimada, & Matsuura, 2002). *C. aggregans* is thermophilic and hence is often found in natural hot springs (Hanada, Hiraishi, Shimada, & Matsuura, 1995a,b; *Hanada* et al.*,* 2002; Jørgensen & Nelson, 1988; Pierson & Castenholz, 1995). *H. aurantiacus* has the ability to perform facultative “wolfpack” predation by excreting hydrolytic enzymes that degrade their prey, therefore its persistence may be due to its unique predation method (Kiss et al., 2011). *P. necessarius* is ubiquitous in freshwater lakes and has strains that are symbiotic as well as free‐living. The free‐living strain is found in abundance in freshwater systems globally and has a reduced genome and limited metabolic flexibility, whereas the symbiotic strains inhabit the ciliate *Euplotes* (Boscaro et al., 2013; Hahn, 2003 *). P. necessarius* cells are small, which are believed to provide predation protection (Hahn, 2003; Hahn, Moore, & Höfle, 1999; Simek et al., 1997;). *P. necessarius* was more abundant downstream (Fig. 3), perhaps indicating a source of this species between the two sites.

*Propionibacterium acnes* was more abundant downstream (Fig. 3). An abundance of *P. acnes* has been identified downstream of Murray Bridge previously in this same river system (Dann, Smith, Jeffries, et al., 2016b) and was suggested to indicate anthropogenic effects from the compact human population within Murray Bridge where waste may have entered into the river via land run‐off, hence supporting our hypothesis.

### 4.4 Marine species abundance in freshwater systems

Despite a clear freshwater community presence and an indication of human input, neither of these could mask a dominant marine community presence. Unicellular photosynthetic *Synechococcus* and *Prochlorococcus* are abundant within the ocean and contribute to a substantial proportion of primary productivity (Sullivan, Waterbury, & Chisholm, 2003; Sullivan, Coleman, Weigele, Rohwer, & Chisholm, 2005; Millard, Clokie, Shub, & Mann, 2004; Mann et al., 2005; Hurwitz, Deng, et al. 2013a; Hurwitz, Hallam, et al. 2013b). Here, we report *Synechococcus* and *Prochlorococcus phage* were the most abundant viral species in this freshwater river system (Fig. 1). The most abundant type strains of *Synechococcus phage* were *S‐SK1, S‐PM2, S‐CBS4*,* S‐SM2,* and *S‐SSM7*. These specific *Synechococcus phage* have been identified in marine datasets, such as the Pacific Ocean Virome (POV) dataset (Hurwitz, Deng, et al. 2013a; Hurwitz, Hallam, et al. 2013b), the Global Ocean Survey (GOS) metagenomes (Frank et al., 2013). The *Synechococcus phage S‐PM2* is a lytic cyanomyovirus capable of infecting multiple marine *Synechococcus* host strains (Mann et al., 2005). The most abundant *Prochlorococcus phage* type strain was *P‐SSM2*. This type strain is marine and has been isolated from oceanic regions and it is capable of infecting three low‐light adapted cyanobacteria host strains (Sullivan et al., 2003, 2005).

Some *Synechococcus* and *Prochlorococcus* phage contain full‐length, conserved photosynthesis genes that originate from cyanobacteria, which are believed to have been acquired via horizontal gene transfer (Sullivan et al., 2003, 2005; Millard et al., 2004; Lindell et al. ). Possession of such genes provides an advantage, such as adaption to light intensity variations and continued repair mechanisms after host cell protein synthesis has shut down, therefore allowing continued photosynthetic activity and oxygen evolution, while providing energy to allow extended viral replication (Havaux, Guedeney, Heand, & Grossman, 2003; Millard et al., 2004; Hurwitz, Deng, et al. 2013a; Hurwitz, Hallam et al. 2013b). Phage infection can therefore have a significant effect on phototroph physiology, the evolutionary path of host‐encoded alleles and hence overall biogeochemical cycling (Mann et al. 2003; Millard et al., 2004;. Hurwitz, Deng, et al. 2013a; Hurwitz, Hallam et al. 2013b) found that these cyanobacterial photosynthesis genes from phage hosts cause metabolic reprogramming, which goes on to influence microbial‐driven carbon metabolism in the euphotic to aphotic ocean. Therefore, if the effects of these phage are similar to their roles in marine environments, their abundance will affect microbial metabolic carbon fluxes (Hurwitz, Deng et al. 2013a Hurwitz, Hallam et al. 2013b).

Wilhelm et al. (2006) also found an abundance of marine *Synechococcus* cyanophage in a freshwater system, a Great Laurentian Lake. Their presence was suggested to be due to introduction mechanisms, such as from boat ballast, or from the presence of their natural hosts. The latter agrees with our findings as we found *Synechococcus* and *Prochlorococcus* in the bacterial metagenomic profiles, hence indicating these may be the hosts for the *Synechococcus* and *Prochlorococcus* phage identified (Fig. S6). The presence of these microbial hosts provides evidence for viral propagation in different biomes due to host movement and growth in different environments, as suggested by Sano et al. (2004). Wang et al. (2015) found viruses similar to those that infect marine *Synechococcus* species within a freshwater lake, East Lake in China, using a set of viral family primers. They suggested the high similarity to marine viruses was due to limited genome reporting of freshwater cyanophage, but also suggested the existence of common ancestors between marine and freshwater environments (Wang et al., 2015). Therefore, cyanophage, as was suggested by Wilhelm et al. (2006), specifically *Synechococcus* and *Prochlorococcus* phage, as well as their potential *Synechococcus* and *Prochlorococcus* hosts, may be as important in freshwater systems as they have been proven to be in marine environments. Our results provide strong support for this hypothesis.

In addition to *Synechococcus* and *Prochlorococcus,* other marine species were identified in the bacterial metagenomic profiles but at low abundances: *Marinobacter hydrocarbonoclasticus, marine Actinobacterium PHSC20C1*,* Bacteriovorax marinus*,* Idiomarina baltica,* and *Idiomarina loihiensis* (Table S2). *M. hydrocarbonoclasticus* is a halotolerant marine bacterium that is capable of degrading a range of aromatic or aliphatic hydrocarbons (Gauthier et al., 1992). *I. loihiensis* is a halophilic bacterium that inhabits hydrothermal vents and relies primarily on amino acid catabolism (Donachie, Hou, Gregory, Malahoffand, & Alam, 2003; Hou et al., 2004; Wagner, 2005). It was suggested this bacterium accesses amino acids via proteinaceous particles occurring in deep sea hydrothermal vent waters (Hou et al., 2004). The presence of these vent bacteria in fresh surface water supports the principle of “*everything is everywhere*, but, *the environment selects*” by Baas‐Becking (1934) arguing microbial species are distributed globally, however, specific environmental conditions will allow specific species to thrive (Beijerinck, 1913; De Wit & Bouvier, 2006). Marine actinobacteria, such as *Marine actinobacterium PHSC20C1,* are closely related to nonmarine actinobacteria and have been suggested to have been recently independently introduced to marine environments (Penn & Jensen, 2012). *B. marinus* is a halophilic bacterium that inhabits high salinity environments (Crossman et al., 2013). This bacterium preys on gram‐positive bacteria and lives in two phases: a highly motile phase where they search for their prey and a growth phase after they gain entry inside the interperiplasmic space of their prey (Baer, Ravel, Piñeiro, Guether‐Borg, & Williams, 2004; Crossman et al., 2013).

What determines the dispersal patterns of microbial communities remains unknown, however, it has been hypothesized that microbial distributions are random, “everything is everywhere” and the environment selects, historical events alone or historical events in combination with current environmental conditions determine microbial spatial variation (Martiny et al., 2006). Here, we provide explanations for why microbial species that had the highest similarity to marine species were present in high abundance in this freshwater system.

First, the presence of marine species could be a result of marine‐freshwater transitions. Previously, such transitions were considered infrequent due to a lack of close relatedness between marine and freshwater microbial phylogenies (Logares et al., 2009). However, microbes have high reproductive rates, large population sizes and the ability to widely disperse, which could support such environmental transitions (Logares et al., 2009). If this is indeed the case, this suggests transitions between marine and freshwater environments may be more frequent than previously assumed.

Alternatively, in regards to historical events, the presence of marine species may be an indication of marine microbial relict communities. Marine transgressions occurring during the Oligocene to Pliocene led to seas flooding the eastern coast of South Australia and subsequently creating marine organism relicts and high concentrations of salt within the sediments and limestone of the river bed (Brown et al., 1968; Kingham, 1998; Zhisheng et al., 1986). Therefore, the abundance of marine species observed may be due to the saline history of this river system.

The effects of increasing salinity from drought or groundwater influences and/or the introduction of marine species via upstream saline regions from increased water flows and releases to the South Australian regions of the Murray River in previous years (DEWNR, 2014a,b), may have led to the selection of microbial species that are saline‐tolerant. This is in line with “*everything is everywhere*, but, *the environment selects*” (Baas‐Becking, 1934; Beijerinck, 1913; De Wit & Bouvier, 2006) and is supported via a previous study on the River Murray that isolated bacterial lineages, specifically LD12 and ACK‐M1, which are believed to have the ability to adapt to varying salinity levels (Zwart, Crump, Agterveld, Hagen, & Han, 2002; Brown et al., 2012; Dann, Smith, Jeffries, et al., 2016b). Increasing salinity levels in this regulated river system from saline groundwater influences are an ongoing concern in the Murray River (Cook, Jollyand, & Leaney, 2001; Goss, 2003; Van Dijk, Hairsine, Arancibia, & Dowling, 2007).

As previously mentioned by Wang et al. (2015), the viral and bacterial species identified here show the highest similarity to marine strains. However, as freshwater metagenomics studies lag behind their marine counterpart, the high similarity to species from marine environments may be because more closely related species within freshwater environments are yet to be sequenced (Wang et al., 2015). Specifically, as only three unique strains of *Pandoravirus* have currently been isolated and these isolates rely on *Acanthamoeba sp*. hosts, which are found in marine and freshwater environments, the abundance of *Pandoravirus salinus* may not be definitive evidence for the presence of viruses from marine origin (Antwerpen et al., 2015; Scheid, 2016).

#### 4.4.1 Giant viruses in freshwater systems

Giant viruses, specifically *Pandoravirus salinus, Mimivirus A*,* Cafeteria roenbergensis virus BV‐PW1, Pithovirus sibericum,* and *Pandoravirus dulcis,* were identified in this freshwater river system (Fig. 1). Although, giant virus species have been isolated from river systems previously, these river species were not abundant in this system (Thomas et al. 2011; Colson et al., 2012; Yoosuf et al., 2013; Campos et al., 2014).

The second most abundant giant virus, *Mimivirus A*, also termed “microbe mimicking virus”, was the first giant virus to be discovered and was isolated from an *Acanthamoeba* in a water tower in Bradford, United Kingdom (Fischer, Allen, Wilson, & Suttle, 2010; La Scola et al., 2008; Tsai, 2011; Wodarz, 2013). Ghedin and Claverie (2005) showed the closest homologs for 43% of the core genes in *Mimivirus* were present in the Sargasso Sea (Claverie et al., 2009), whereas Monier et al. (2008) found relatives of the *Mimivirus* were the second most abundant group of marine viruses within the Sargasso Sea (Claverie et al., 2009). Here, we suggest *Mimivirus* may also be an abundant member of freshwater systems. The exact ecological role of these giant viruses remains unknown, however, the results provide more support for the prevalence of giant viruses in freshwater river systems globally.

#### 4.4.2 Persistence of bacterial and viral species

The similarity in bacterial and viral species upstream and downstream indicates persistence. Previously, persistence of bacterial genotypes has been suggested to be due to the continuum of fluvial systems preventing the restriction of microbes to particular locations (McArthur & Tuckfield, 1997). Studies by Wise, Shimkets, and McArthur (1995) and McArthur, Leff, and Smith (1992) showed the adaptation of *Burkholderia (Pseudomonas) cepacia* and *Pseudomonas pickettii,* to patchy microenvironments over 5 km and 3.5 km distances. Dann, Smith, Jeffries, et al. (2016b) identified bacterial genotype persistence upstream and downstream of this same environment over a similar scale, suggesting these persistent genotypes were the dominant archetypal taxa, which would therefore impact system function.

The presence of a persistent freshwater community, human input indicators and dominant marine community, indicate an environment where species addition leads to higher diversity rather than increased selection or exclusion. This river community is therefore cosmopolitan, as was suggested by Beijerinck (1913), or adoptive, rather than endemic, where limited diversity is locally generated, such as what is observed in the Amazon or Great Barrier Reef (Bellwood, Hughes, Folke, & Nyström, 2004; Brooks et al., 2002). This supports Baas‐Becking (1934), where there is environmental selection of cosmopolitan rare taxa, and highlights whether there are other locations that accumulate species from multiple different environments.

#### 4.4.3 Functional profile

This environment was dominated by phage‐related processes, with phage, prophage, transposable elements and plasmids more abundant downstream, and phage protein significantly higher downstream at the function level (Fig. 5). As phage are known to dominate the viral community, this would explain the high abundance of phage‐associated processes (Breitbart et al., 2002; Hendrix, 2002).

In addition, sequences associated with membrane transport, carbohydrates, amino acids, and protein metabolism were also abundant (Fig. 4). Previously, Dinsdale et al. (2008) found a high percentage of sequences associated with carbohydrate metabolism within microbial and viral metagenomes from a range of environments. However, it was suggested that there are discriminatory metabolic pathways across environments, with these pathways indicating the processes that are important for the growth and survival of microbial communities within the specific environment studied (Dinsdale et al., 2008). Therefore, carbohydrate, amino acid, and protein metabolism may be the most important core functions within this freshwater river system.

#### 4.4.4 Rank abundance of freshwater viral populations

Rank abundance graphs of the viral genotypes were used to illustrate contrasting levels of species richness, and to determine the mathematical function that best described the community structure of the viruses upstream and downstream (Edwards & Rohwer, 2005; Magurran, 2004). In this study, the upstream and downstream community had a generalized Pareto distribution with no significant difference in function and slope (Fig. 2). Generalized Pareto distributions are power law distributions, which have been found previously in viral genotype communities, specifically phage (Angly et al., 2005; Edwards & Rohwer, 2005; Hoffmann et al., 2007). Power law distributions within viral genotypes are suggestive of two different ecological mechanisms. The first mechanism involves different viral genotypes competing for the same microbial host, followed by stochastic viral behavior that leads to one viral genotype infecting additional hosts. Infection of additional hosts then leads to increased abundance of this viral genotype via each lytic cycle, therefore resulting in increased fitness for infection and replication while the other limited‐host genotypes remain rare (Edwards & Rohwer, 2005). The second mechanism involves a viral genotype that infects a single microbial species. The microbial hosts compete for the same energy source and randomly one host obtains more food and hence divides faster, hence leading to a power law distribution of the microbial hosts. Once viruses begin infecting these microbial hosts, this results in a subsequent power law distribution for the viruses (Edwards & Rohwer, 2005). In both instances, this leads to the power law distribution of viral communities due to a combination of connected, exponential mechanisms and are examples of the “rich‐get‐richer” idiom (Edwards & Rohwer, 2005). “Rich‐get‐richer” mechanisms include predator‐prey models, where phage diversity is strongly linked to the diversity and structure of the coexisting microbial community (Angly et al., 2005). Therefore, the same mathematical function between the viral metagenomes may reflect similarities in the microbial community at each site, which was observed in the bacterial metagenomics profiles.

## 5 Conclusions

Here, we report an abundance of marine *Synechococcus* and *Prochlorococcus* phage, as well as the marine giant virus *Pandoravirus salinus* in a freshwater river system. The abundance of marine species may be attributed to marine‐freshwater transitions, marine microbial relicts from the marine history of this river system, or a result of environmental selection from the effects of increasing salinity from drought or groundwater influences and/or the introduction of marine species via upstream saline regions from increased water flows and releases.

As microbial composition affects heterotrophic and autotrophic production, carbon dioxide respiration and decomposition, and the cycling of critical nutrients, the abundance of marine viruses in this freshwater river suggests marine microbial species may be as important in the function of this particular freshwater ecosystem as they are for marine.

Giant viruses, namely *Pandoravirus salinus, Mimivirus A*,* Cafeteria roenbergensis virus BV‐PW1, Pithovirus sibericum,* and *Pandoravirus dulcis* were present, with *Pandoravirus salinus* and *Mimivirus A* having high abundance upstream and downstream, therefore suggesting giant viruses may be important role players in the function of this freshwater ecosystem. The abundance of *Human herpes 6A* was attributed to anthropogenic effects on this river system and suggests this could serve as another traceable indicator for human impact within freshwater river systems.

An abundance of *Chloroflexus aggregans, Herpetosiphon aurantiacus,* and *Polynucleobacter necessarius* revealed a bacterial community that is thermophilic, capable of “wolfpack” predation or capable of two life strategies: free‐living or symbiotic. This suggests this river system hosts a range of diverse and potentially hardy microbial species that are perhaps able to persist regardless of the water flow and salinity fluctuations observed in this river system.

## Funding Information

This research was supported by a CSIRO Land and Water Top‐Up PhD Scholarship (LD), a CSIRO consumables grant (LD), an APA to LD, Centre for Excellence for Environmental decisions, and Australian Research Council grants, www.arc.gov.au, LP‐130100508 and DP‐150103018 to JGM.

## Conflicts of Interest

The authors declare no conflicts of interest.
