# Supplementary material for: Marine and giant viruses as indicators of a marine microbial community in a riverine system
Source: Microbiologyopen. 2016 Aug 9;5(6):1071–84. doi: 10.1002/mbo3.392 (PMC5221468; doi:10.1002/mbo3.392)
Supplement: Supplementary file 1 [file MBO3-5-1071-s001.docx]

**Supplementary Information**

**
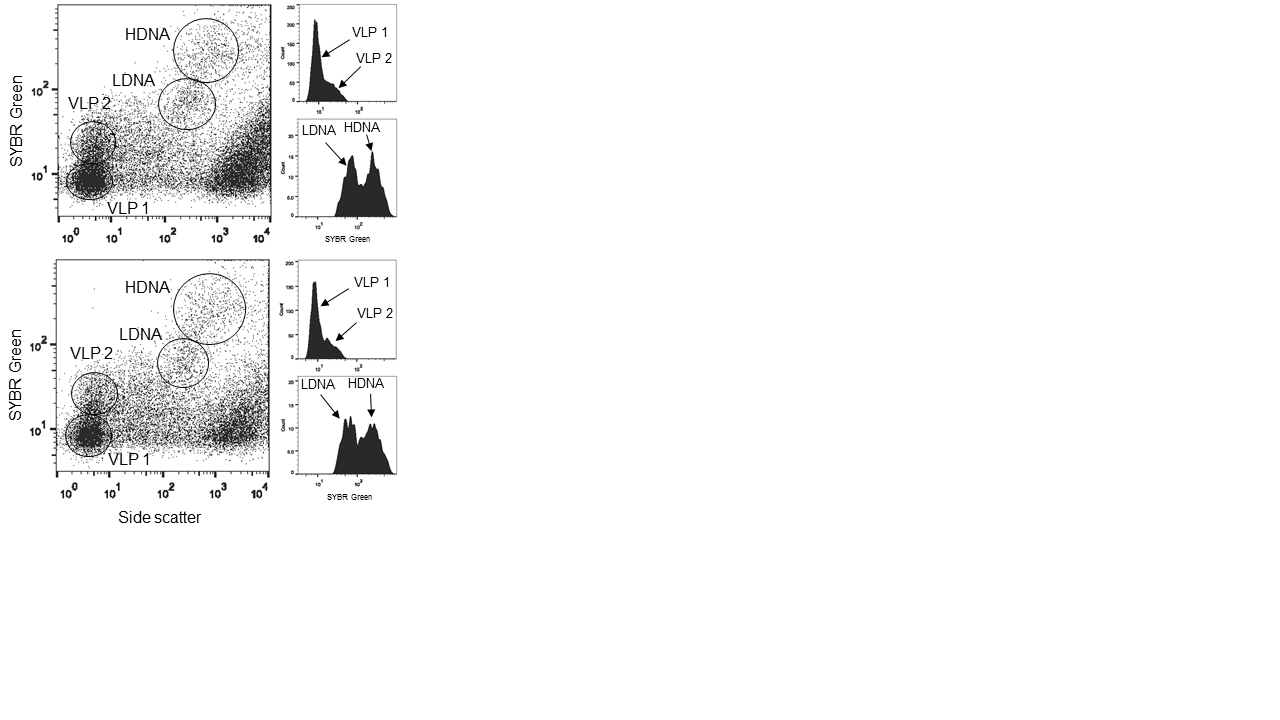
**

**Figure S1.** Flow cytometric cytograms of side-scatter and SYBR Green fluorescence and histograms of SYBR Green fluorescence showing two VLP subpopulations, VLP 1 and VLP 2, and two prokaryotic subpopulations, LDNA and HDNA, **(A)** upstream and **(B)** downstream.

.

**Figure S2.** Average abundance of viral families upstream and downstream. For clarity, only families representing ≥ 0.1% average abundance are shown. Viral families determined via 49-kmer contigs blasted against the NCBI nucleotide database using tBLASTx.

**Figure S3**. Average abundance of viral genera upstream and downstream. For clarity, only genera representing ≥ 0.1% average abundance are shown. Viral genera determined via 49-kmer contigs blasted against the NCBI nucleotide database using tBLASTx.

**Figure S4.** Average abundance of bacterial phyla upstream and downstream. Phyla determined via the MG-RAST server pipeline using the SEED database. For clarity, only phyla contributing to > 0.1% average abundance are shown.

**Figure S5.** Average abundance of bacterial families upstream and downstream. Families determined via the MG-RAST server pipeline using the SEED database. For clarity, only families contributing to > 1% average abundance are shown.


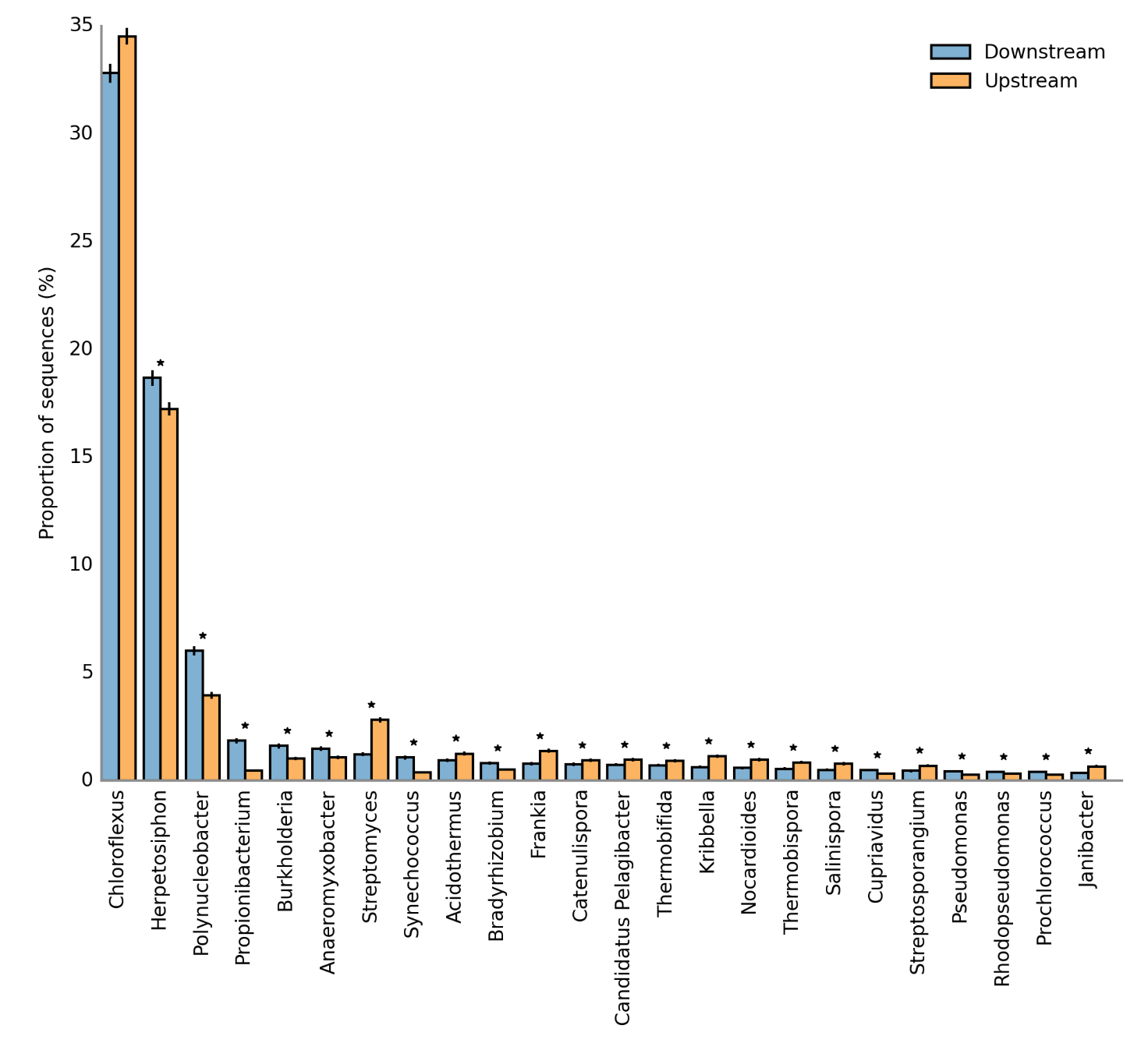


**Figure S6.** Comparison of the proportion of sequences for each bacterial genus upstream and downstream. Bacterial genera determined via the MG-RAST server using the SEED non-redundant database. Profile bar plot produced in STAMP using Fisher exact test with Benjamini-Hochberg FDR multiple test correction to calculate the p-value. The p-value threshold was 0.05. Asterisks indicate significant differences between upstream and downstream taxa. The 95% confidence intervals were calculated using the Newcombe-Wilson method.

**
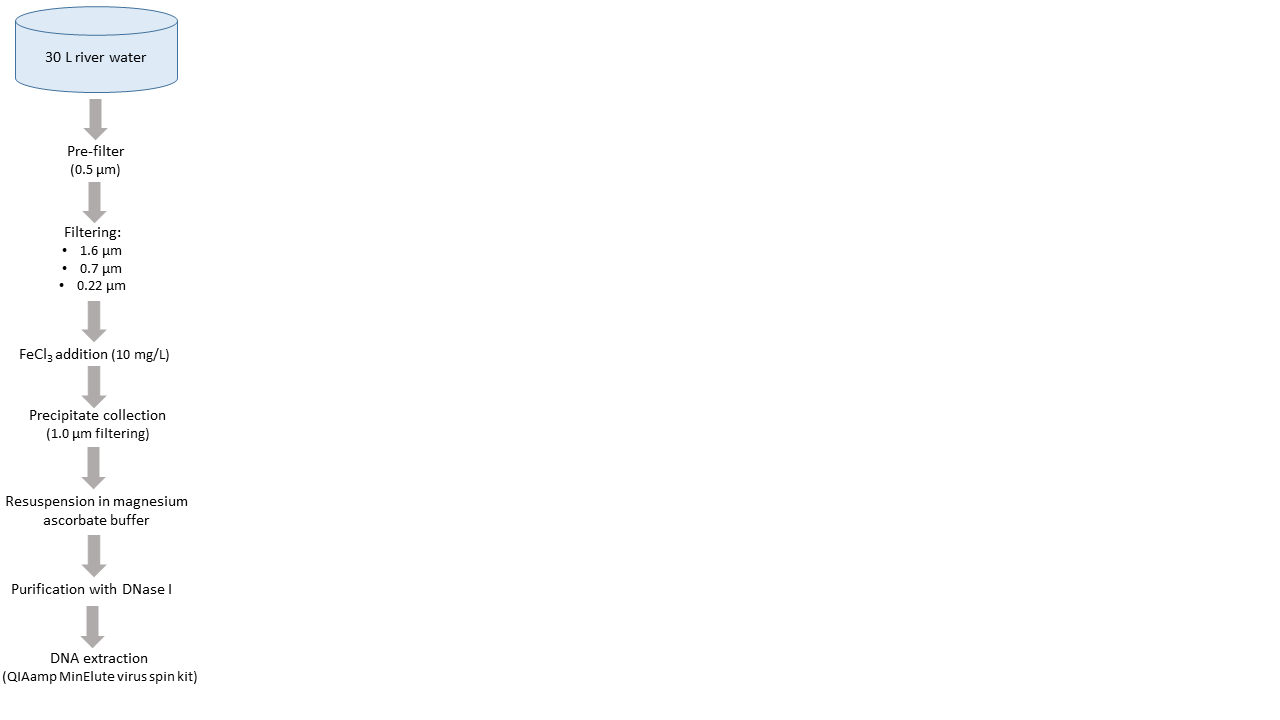
**

**Figure S7.** Workflow showing the concentration and purification of freshwater viral communities.

**Table S1.** Resulting power law values for maximum likelihood testing of upstream and downstream sites. N = number of genotypes.

| **Site** | **n** | **xmin** | **alpha** | **KS-statistic** | **KS critical value** |
| --- | --- | --- | --- | --- | --- |
| Downstream | 682 | 8 | 1.66 | 0.056 | 0.059 |
| Upstream | 508 | 7 | 1.71 | 0.069 | 0.072 |

**Table S2.** Average abundances of bacterial species present upstream and downstream. For clarity, only species representing ≥ 0.10% average abundance are shown.

| **Species** | **Downstream** | | **Upstream** | |
| --- | --- | --- | --- | --- |
| *Streptomyces coelicolor* | 0.00% | 1.01% | |  |
| *Propionibacterium acnes* | 1.85% | 0.46% | |  |
| *Haemophilus influenzae* | 0.47% | 0.01% | |  |
| *Spirosoma linguale* | 0.00% | 0.49% | |  |
| *Nitrosomonas eutropha* | 0.04% | 0.56% | |  |
| *Azotobacter vinelandii* | 0.03% | 0.52% | |  |
| *Gloeobacter violaceus* | 0.00% | 0.40% | |  |
| *Shigella sonnei* | 0.04% | 0.54% | |  |
| *Cytophaga hutchinsonii* | 0.00% | 0.37% | |  |
| *Polynucleobacter necessarius* | 6.01% | 3.96% | |  |
| *Sulfurimonas denitrificans* | 0.00% | 0.33% | |  |
| *Dyadobacter fermentans* | 0.27% | 0.00% | |  |
| *Synechococcus sp. RS9917* | 0.23% | 0.00% | |  |
| *Xylanimonas cellulosilytica* | 0.00% | 0.24% | |  |
| *Loktanella vestfoldensis* | 0.20% | 0.00% | |  |
| *Dechloromonas aromatica* | 0.15% | 0.59% | |  |
| *Paenibacillus larvae* | 0.24% | 0.02% | |  |
| *Chloroflexus aurantiacus* | 1.49% | 0.83% | |  |
| *Blastopirellula marina* | 0.39% | 0.11% | |  |
| *Kribbella flavida* | 0.63% | 1.14% | |  |
| *Frankia sp.* | 0.25% | 0.60% | |  |
| *Acinetobacter baumannii* | 0.31% | 0.09% | |  |
| *Burkholderia pseudomallei* | 0.90% | 0.48% | |  |
| *Chloroflexus aggregans* | 31.24% | 33.62% | |  |
| *Candidatus Protochlamydia amoebophila* | 0.31% | 0.09% | |  |
| *Actinobacillus pleuropneumoniae* | 0.05% | 0.24% | |  |
| *Streptomyces avermitilis* | 0.69% | 1.13% | |  |
| *Nocardioides sp. JS614* | 0.58% | 0.97% | |  |
| *Janibacter sp. HTCC2649* | 0.36% | 0.67% | |  |
| *Kineococcus radiotolerans* | 0.29% | 0.56% | |  |
| *Candidatus Solibacter usitatus* | 0.36% | 0.16% | |  |
| *Methylacidiphilum infernorum* | 0.26% | 0.10% | |  |
| *Salinispora tropica* | 0.35% | 0.62% | |  |
| *Herpetosiphon aurantiacus* | 18.63% | 17.20% | |  |
| *Nitrosospira multiformis* | 0.23% | 0.08% | |  |
| *Anaeromyxobacter sp. Fw109-5* | 1.43% | 1.03% | |  |
| *Thermobispora bispora* | 0.55% | 0.85% | |  |
| *Frankia sp. EAN1pec* | 0.22% | 0.41% | |  |
| *Albidiferax ferrireducens* | 0.11% | 0.25% | |  |
| *Streptosporangium roseum* | 0.45% | 0.69% | |  |
| *Acidothermus cellulolyticus* | 0.95% | 1.25% | |  |
| *Escherichia coli* | 0.28% | 0.15% | |  |
| *Renibacterium salmoninarum* | 0.14% | 0.27% | |  |
| *Bradyrhizobium sp. BTAi1* | 0.28% | 0.16% | |  |
| *Azorhizobium caulinodans* | 0.30% | 0.18% | |  |
| *Bacillus licheniformis* | 0.21% | 0.11% | |  |
| *Nocardia farcinica* | 0.19% | 0.32% | |  |
| *Candidatus Pelagibacter ubique* | 0.75% | 0.97% | |  |
| *Thermobifida fusca* | 0.72% | 0.92% | |  |
| *Rhodococcus jostii* | 0.24% | 0.36% | |  |
| *Robiginitalea biformata* | 0.21% | 0.12% | |  |
| *Catenulispora acidiphila* | 0.76% | 0.96% | |  |
| *Prochlorococcus marinus* | 0.41% | 0.29% | |  |
| *Opitutus terrae* | 0.20% | 0.31% | |  |
| *Geodermatophilus obscurus* | 0.35% | 0.48% | |  |
| *Nocardiopsis dassonvillei* | 0.22% | 0.32% | |  |
| *Saccharopolyspora erythraea* | 0.23% | 0.33% | |  |
| *Pedobacter heparinus* | 0.28% | 0.38% | |  |
| *Pelotomaculum thermopropionicum* | 0.29% | 0.20% | |  |
| *Kytococcus sedentarius* | 0.13% | 0.20% | |  |
| *Janthinobacterium sp. Marseille* | 0.22% | 0.15% | |  |
| *Cupriavidus metallidurans* | 0.24% | 0.17% | |  |
| *Actinosynnema mirum* | 0.24% | 0.33% | |  |
| *Cupriavidus pinatubonensis* | 0.23% | 0.16% | |  |
| *Streptomyces griseus* | 0.32% | 0.42% | |  |
| *Mesorhizobium loti* | 0.20% | 0.13% | |  |
| *Desulfotomaculum reducens* | 0.20% | 0.14% | |  |
| *Rhodopseudomonas palustris* | 0.41% | 0.32% | |  |
| *Mycobacterium smegmatis* | 0.14% | 0.20% | |  |
| *Nakamurella multipartita* | 0.19% | 0.25% | |  |
| *Bradyrhizobium japonicum* | 0.36% | 0.29% | |  |
| *Thermomonospora curvata* | 0.53% | 0.62% | |  |
| *Leifsonia xyli* | 0.17% | 0.22% | |  |
| *Ralstonia solanacearum* | 0.18% | 0.23% | |  |
| *Frankia alni* | 0.31% | 0.37% | |  |
| *Saccharomonospora viridis* | 0.19% | 0.24% | |  |
| *Delftia acidovorans* | 0.25% | 0.29% | |  |
| *Streptomyces scabiei* | 0.22% | 0.25% | |  |
| *Stackebrandtia nassauensis* | 0.23% | 0.20% | |  |
| *Cellulomonas flavigena* | 0.22% | 0.25% | |  |
| *Chitinophaga pinensis* | 0.84% | 0.82% | |  |
| *Clavibacter michiganensis* | 0.22% | 0.21% | |  |
| *marine actinobacterium PHSC20C1* | 0.21% | 0.21% | |  |
